# Multilevel determinants of medication preferences for opioid use disorder among criminal-legal-involved populations: Insights from a scoping review

**DOI:** 10.1016/j.dadr.2025.100395

**Published:** 2025-11-16

**Authors:** Amelia Bailey, Jaclyn M.W. Hughto, Claudia Stagoff-Belfort, Shira I. Dunsiger, Rosemarie A. Martin

**Affiliations:** a Center for Alcohol and Addiction Studies, Brown University School of Public Health, Providence, RI 02903, USA; b Department of Behavioral and Social Sciences, Brown University School of Public Health, Providence, RI 02903, USA; c Center for Health Promotion and Health Equity, Brown University School of Public Health, Providence, RI 02903, USA; d Department of Epidemiology, Brown University, Providence, RI 02903, USA; e Department of Psychiatry and Human Behavior, Brown University, Providence, RI 02903, USA; f Department of Biostatistics, Brown University, Providence, RI 02903, USA; g Division of Preventive and Behavioral Medicine, UMass Chan Medical School, Worcester, MA, 01655, USA

**Keywords:** Opioid use disorder (OUD), Medications to treat opioid use disorder (MOUD), Preferences, Attitudes, Criminal-legal system

## Abstract

**Background:**

Individuals with opioid use disorder (OUD) hold varying perceptions of the three medications to treat OUD (MOUD). Data on these perceptions among those with criminal-legal involvement is limited. To optimize MOUD service delivery, especially in criminal-legal settings, it is essential to explore the preferences and attitudes of those with legal involvement.

**Methods:**

We conducted a scoping review of literature published via PubMed, Embase, Web of Science, Scopus, PsycInfo, and SocINDEX (January 1, 2014-September 17, 2024). Three-stage screening process was employed by two reviewers: title/abstract (n = 2085 articles), full-text (n = 88), and data extraction of 44 articles included in the final sample. Content analysis was used to understand preferences/attitudes toward MOUD and related influential factors.

**Results:**

Factors that influenced MOUD preferences/attitudes were clustered in positive (n = 39 articles; i.e., like), negative (n = 38; i.e., dislike), or mixed (n = 20; both positive and negative) valences. Methadone was the most referenced (n = 30), with more articles noting negative attitudes than positive. Fewer studies focused on oral buprenorphine (n = 18), with balanced positive and negative views. Seven articles on injectable MOUD highlighted mostly positive attitudes. Factors shaping preferences/attitudes spanned from individual to structural levels. Common factors associated with negative preferences included MOUD program rules, side or adverse effects, and drug-free ideology. Positive preferences were often engendered by flexible MOUD delivery and beliefs about MOUD. Influential factors differed by MOUD type.

**Conclusions:**

Preferences/attitudes toward MOUD among criminal-legal-involved populations are shaped by intersecting multilevel determinants and differ by MOUD type. Identified factors might serve as intervention targets to better meet individuals’ needs.

## Highlights

• Reviewed opioid use disorder medication attitudes for those with legal history
• 44 included studies describe positive and negative attitudes by medication type
• Methadone most often referenced type, with attitudes often negative
• Buprenorphine attitudes both negative and positive, and injectables positive
• Multilevel factors influence views (e.g., program rules), vary by medication type

## 1 Background

The opioid overdose epidemic has contributed to substantial social harms, morbidity, and mortality (Judd et al., 2023). Buprenorphine, methadone, and naltrexone are the three FDA-approved, evidence-based medications for opioid use disorder (MOUD) (National Academies of Sciences and Medicine, 2019), yet these medications are underutilized, particularly in the criminal-legal system (Berk et al., 2025b, Scott et al., 2021). Federal efforts attempt to reduce barriers to accessing MOUD and to enhance the quality of care (e.g., Substance Abuse and Mental Health Services Administration 2022). However, stark barriers remain across the individual (e.g., internalized stigma), community (e.g., access to treatment), and structural (e.g., legal policies) levels of the social-ecological model (Bronfenbrenner, 1977). These barriers are especially pronounced for those with current or previous criminal-legal system involvement (Jalali et al., 2020, Mackey et al., 2020, Russell et al., 2022b).

A myriad of factors place individuals with criminal-legal involvement at disproportionate risk for poor health, such as insufficient healthcare during incarceration and societal stigma associated with incarceration (National Academies of Sciences and Medicine, 2019, Sugarman et al., 2020). Innovative carceral-based programs provide access to MOUD, yet these programs are often fledgling with restrictive access criteria for incarcerated persons with opioid use disorder (OUD) (Clarke et al., 2018, Macmadu et al., 2020, Scott et al., 2021). As more carceral facilities begin to provide or scale up MOUD access and adjacent criminal-legal settings aim to enhance access to such care (e.g., warm hand-offs from courts), it is necessary to identify the factors that influence MOUD engagement among people with OUD who are involved in the criminal-legal system.

Prior research has investigated individuals’ perceptions of MOUD within and outside of the criminal-legal system. When provided access to all types of MOUD, individuals with OUD often perceive differences between the two most common types of MOUD utilized, buprenorphine and methadone, in the community (Gryczynski et al., 2013) and during incarceration (Kaplowitz et al., 2022b). The perceived acceptability of MOUD types varies by the individual and factors that uniquely influence them. The social-ecological model can help us understand how these influential factors spanning multiple levels - individual, interpersonal, community, and structural - interact to shape preferences for and attitudes toward MOUD among those with criminal-legal involvement (Bronfenbrenner, 1977, Jalali et al., 2020, McLeroy et al., 1988, Russell et al., 2022b, Stokols, 1992). For example, individual-level recovery goals and identity-related factors (race, ethnicity, and gender), and structural-level stigma may impact treatment decisions (Gryczynski et al., 2013, Kaplowitz et al., 2022b, Scott et al., 2021).

It remains unclear how and why MOUD *preferences* differ as influential factors evolve, including structural changes such as in illicit drug supply and in treatment policies like expanded take-home dosing. Importantly, the widespread presence of fentanyl in the US drug supply may critically impact MOUD experiences (Ciccarone, 2021). Exposure to fentanyl long-term may increase initial treatment dosage (Chandra et al., 2021), complicate induction on buprenorphine (Comer and Cahill, 2019, Shearer et al., 2022, Varshneya et al., 2022), and reduce buprenorphine and methadone adherence and retention (Hochstatter et al., 2022, Socias et al., 2022). Given these effects, it is necessary to understand the breadth of patient experiences in the current era of the drug supply. Currently, there is no existing literature synthesis on how MOUD preferences are informed by multilevel factors, spanning individual to structural, limiting the ability to specify what patients desire from their treatment (and why) and to address needs and gaps in care that might relate to treatment preferences at multiple levels. To ensure that individuals access OUD treatment that is beneficial via more equitable systems of care, it is critical to understand how perceptions of MOUD are shaped for individuals who are incarcerated.

A scoping review by Cioe et al. (2020) identified 152 studies examining OUD patient and provider perspectives between 1949 and 2019. Structural-level stigma toward MOUD was present across most studies and there was a general lack of knowledge about MOUD among patients and providers, which was theorized to contribute to poorer provision of care and influence selection of medication (Cioe et al., 2020). Importantly, the review did not investigate these perspectives among individuals with criminal-legal involvement histories, therefore limiting the transferability to this unique population. Additionally, another review sought to examine the role of preferences in studies of MOUD effectiveness for individuals involved with the criminal-legal system (n = 27 studies). Only 6 of the included studies incorporated MOUD preferences into study design (i.e., investigated how participant MOUD choice impacted outcomes) or measured preferences to inform the interpretation of findings (Puglisi et al., 2019). Given these gaps, synthesized data on preferences for MOUD among populations with criminal-legal involvement is warranted.

In the present study, we systematically conducted a scoping review of empirical literature to assess the current knowledge base on attitudes toward and preferences for MOUD among criminal-legal-involved populations to identify future intervention targets to better meet patient preferences and enhance treatment congruence. As such, we aimed to summarize (a) characteristics of studies that assess preferences/attitudes, (b) characteristics of the participants sampled in these studies, (c) stated preferences for and attitudes toward MOUD in these samples, and (d) factors that influence these preferences across social-ecological levels. This review will inform community partners, treatment providers, public health practitioners, and evaluators who work in the space of treatment provision for OUD within and around criminal-legal settings.

## 2 Method

This review documents the existing evidence since 2014, when fentanyl emerged in the US (Ciccarone, 2021). Mirroring the changes to the drug supply, significant changes to MOUD provision in carceral settings also occurred since 2014 (Clarke et al., 2018). The methods and results follow the Preferred Reporting Items for Systematic Review and Meta-Analysis criteria modified for scoping reviews (PRISMA Sc-R) (Tricco et al., 2018) (Appendix A) and are informed by the framework set forth by Arksey and O'Malley (2005). This scoping review protocol was published on the Open Science Foundation (OSF).

### 2.1 Information sources and search strategy

In consultation with a public health research librarian, an initial systematic search was developed in PubMed (Appendix B) using a mixture of relevant MeSH terms and common and uncommon terms used in the field corresponding to the search topics. Population, outcome, and intervention of interest included: medications to treat opioid use disorder, the criminal-legal system, and preferences/attitudes. This search was translated to the appropriate terms for five additional databases using the Polyglot Systematic Review Accelerator for keywords (Clark et al., 2020) and MeSH terms relevant to each database. The systematic search of PubMed, PsycInfo, SocINDEX, Embase, Web of Science, and Scopus was conducted on September 17, 2024.

### 2.2 Eligibility criteria

Inclusion criteria: Articles were included in this review if published before September 17, 2024, and since January 1, 2014. Articles were included when they fit the following inclusion criteria: adults 18 years or older; with OUD; with present or former criminal-legal involvement (i.e., arrest, incarceration); and their preferences for or attitudes toward MOUD were described. All study designs were included.

We defined preference as a liking of one option over other alternative(s) (or not liking MOUD as a whole). Attitude was defined as a settled way of feeling about something (e.g., expressing a negative or positive sentiment about MOUD) (Oxford University Press, n.d.). Patient preferences for a given healthcare treatment are based on a complex set of thoughts and past experiences (Casper and Brennan, 1993). At a high level, this scoping review sought to understand how treatment decisions are made and what factors guide these decisions. As such, synonyms for “preference” and “attitudes”, and related keywords were also considered, including but not limited to: perspective, interest, intention, decision, choice, and selection.

Exclusion criteria: Studies were excluded when only patient utilization of MOUD was discussed (i.e., % of patients received methadone). Articles were excluded if non-empirical (i.e., commentaries), published before 2014, not published in English, or if scoping, systematic, or another type of review.

### 2.3 Selection process

The resulting articles were uploaded into Covidence for article review and data extraction (Veritas Health Innovation, 2023). Two reviewers (first and third authors) examined titles/abstracts against the inclusion criteria and excluded articles that clearly did not fit the inclusion criteria. The remaining articles were moved to the next round of review: full texts for each article were uploaded into Covidence and read through by the independent reviewers. Articles were excluded if they did not meet the inclusion criteria. The bibliography of each remaining article was examined for any articles that may fit the inclusion criteria for the present review. This two-round screening process is consistent with established scoping review standards (Arksey and O'Malley, 2005, Peters et al., 2015, Tricco et al., 2016).

### 2.4 Data extraction

Two independent reviewers (first and third authors) systematically extracted the following from each article into a Covidence template: study publication and declarations, study aim, data collection details, sample characteristics, involved criminal-legal intercept (consolidated as police interaction/pre-arrest, arrest, court, jail, prison, post-release period, parole or probation, and other) (Munetz and Griffin, 2006), MOUD preferences/attitudes, and factors impacting preferences/attitudes. For the latter two items, the content was extracted in the form of narrative summaries created by the reviewers (i.e., independently created condensed passages that relied on key phrases used in the given article). Reviewers kept a log during the extraction process; these notes were used to confer with the other reviewer. One reviewer (first author) identified discrepancies between reports. In cases of disagreement, the reviewers consulted the original text and discussed their differing interpretations until they reached a consensus.

#### 2.4.1 *Data abstraction*

Our examination of these factors and presentation of findings in this review were informed by previous scoping reviews that also abstracted social-ecological factors of influence (Garney et al., 2021, Meehan et al., 2023). We replicated a similar process, utilizing directed qualitative content analysis to condense and abstract the narrative summaries into unidimensional concepts (e.g., social-ecological factor) and to determine the frequency of these concepts across the primary articles (Assarroudi et al., 2018, Kleinheksel et al., 2020, Kondracki et al., 2002).

Specifically, we used this method to condense and abstract the narrative summaries in the fields of (a) preferences/attitudes for MOUD type and/or formulation (i.e., oral, sublingual, intramuscular, or subcutaneous injectable) and (b) factors that impacted preferences/attitudes for MOUD. For (a), the narrative summary was deductively coded for the type of MOUD and the valence of the statement. Options for MOUD type included: “MOUD” as a whole (e.g., MOUD, medication-assisted treatment), “methadone,” “orally administered buprenorphine” (i.e., sublingual), “injectable buprenorphine” (long-acting injectable formulations, i.e., intramuscular or subcutaneous) (Kumar et al., 2025), and “naltrexone” (long-acting injectable formulation). Further, the codes for valence included: positive (facilitate access, positive attitudes, or like or interest), negative (bar access, negative attitudes, or dislike or disinterest), or mixed (one statement that expresses positive and negative feelings, or neutral). For (b), inductive codes were generated to identify of factors that influenced preferences/attitudes toward MOUD described in (a), either when these factors were plainly stated (i.e., explicit, manifest; such as “transportation”) or when they required further interpretation (i.e., implicit, latent; such as “drug criminalization”) (Graneheim and Lundman, 2004). These codes were later mapped onto the social-ecological model.

For both processes, two coders (first and third authors) deductively (i.e., for MOUD and for preferences) and inductively (i.e., for influential factors) coded the first five articles in NVivo (Lumivero, 2022) and met to discuss codes. Upon sufficient reconciliation, one coder (first author) single-coded the remaining articles while the alternate coder spot-checked. After completion of inductive coding to identify influential factors, the first author identified the corresponding construct and level within the social-ecological model in which the code resided. The code was then grouped underneath the corresponding “category” (i.e., mutually-exclusive grouping of codes, such as “treatment quality”) that was then nested within a given social-ecological level (i.e., “theme” that links underlying meaning among categories, such as “community-level”) (Graneheim and Lundman, 2004). Finally, the reviewers met to consolidate and refine the findings and format codes in a hierarchical codebook (Appendix D).

### 2.5 Write-up

For study and participant characteristics, data are presented by study and narratively synthesized across studies (Khan et al., 2003). For the content analysis, the frequency of each code is presented by the number of articles that contain a given factor at least once. Codes are presented by valence, and, within valence, the codes are presented by social-ecological level.

## 3 Results

**Fig. 1:**
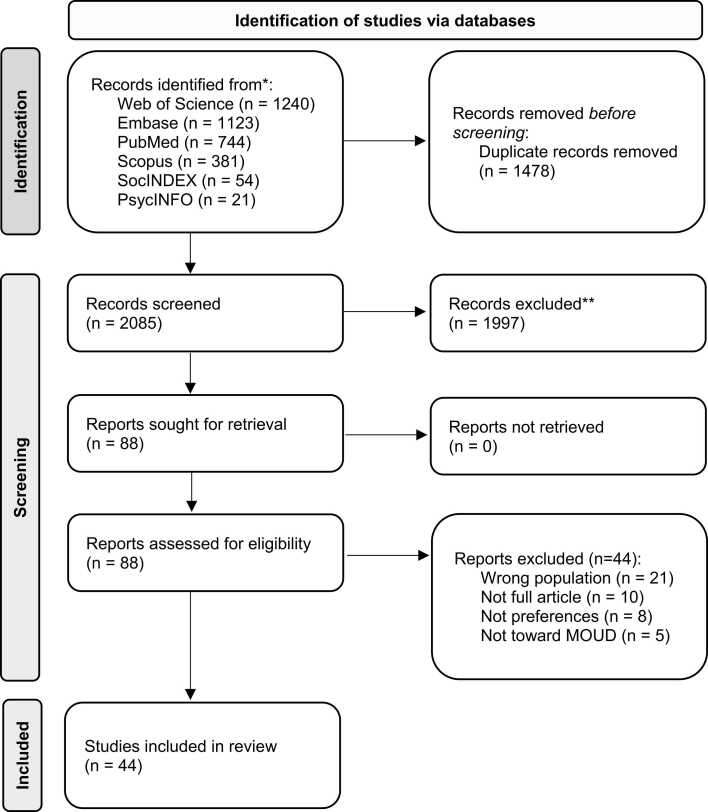
BMJ 2021;372:n71. doi: 10.1136/bmj.n71. Caption: The figure displays the Preferred Reporting Items for Systematic reviews and Meta-Analyses extension for Scoping Reviews 2020 flow diagram.

The database search yielded 2085 articles. Of these articles, 1997 were screened out during title/abstract review. After full-text review of the remaining 88 articles, 44 were included in this review (see Fig. 1).

(a) **PRISMA 2020 flow diagram adapted to this scoping review.** Page MJ, McKenzie JE, Bossuyt PM, Boutron I, Hoffmann TC, Mulrow CD, et al. The PRISMA 2020 statement: an updated guideline for reporting systematic reviews. (b) The diagram, adapted for the present review, shows how many articles were identified through databases, screened by reviewers, and included for this review to result in 44 final articles.

### 3.1 Overall study characteristics

Studies included in this review were most often published in 2022. Around half, 54.5 %, of studies were conducted in the United States (n = 24), followed by Moldova (6.8 %, n = 3), Ukraine (6.8 %, n = 3), and other countries (n = 14). Qualitative inquiry was the most common study design, with 59.1 % of studies employing interviews or focus groups. Fewer studies employed cross-sectional surveys (20.5 %), or multi-method (cross-sectional surveys and qualitative interviews (13.6 %); survey or interview following an experimental study (6.8 %)). Across studies, data collection spanned 12 years (2010–2022), with data most often being collected in 2018. Studies were often published in addiction/substance use-focused journals (72.7 %) (Appendix C for synthesized findings; Table 1 for findings by study).

**Table 1:** Characteristics of the studies in this review that examine preferences toward medications to treat opioid use disorder.

Author (Year)	Location	Aim	Study Design	First Year of Data Collection	Recruitment Setting	Included Population	Sample Size	Criminal-Legal Intercept(s)	MOUD P/A, yes:	MOUD type P/A, yes:	Injectable P/A, yes:
Ascunce Gonzalez et al. (2024)	US	Factors affecting Latine individuals' OAT initiation	Qualitative	2021	Residential treatment; shelter	Adult; Hispanic/Latino/a/e/x; use opioids	21	Jail; Prison; Release; Child protective services	X	X	
Brinkley-Rubinstein et al. (2019)	US	Jail and prison-delivered medication uptake	Qualitative	NR	MOUD program at carceral facility	Adult; OUD; receiving MOUD; received MOUD upon release; attend sessions; English-speaking	40	Jail; Prison; Release	X		
Chappuy et al. (2021)	France	Factors of interest for XR BUP	Cross sectional	2018	Treatment organizations, including prison medical centers	Adult; receiving MOUD; consent; capable of completing questionnaire	317 (TS); 96 (incarcerated sub-sample)	Prison			X
Culbert et al. (2015)	Indonesia	Prevalence, correlates, and social context of injection drug use in prison	Cross sectional study with qualitative	2013	Medical records	Living with HIV; male; fluent in Bahasa Indonesia; consent	102	Prison		X	
Dorgay et al. (2022)	Moldova	Effect of the SBIRT on methadone uptake and retention	Experimental study with survey	2017	Carceral facilities providing methadone	Adult; incarcerated; opioid dependence via ICD−10; < 3 mo release; post-release address near methadone program	121	Jail; Prison		X	
Evans et al. (2023)	US	Perceptions of MOUD diversion in jail; contributors and ways to reduce	Qualitative	2021	At release from incarceration; service settings in community	Previously incarcerated in Massachusetts jail; received MOUD while incarcerated; living in community	38	Jail	X		
Finlay et al. (2020)	US	Barriers to MOUD access	Qualitative	2018	Veteran's program	Adults; English-speaking; consent; past−10 yr opioid use history or OUD; any legal involvement	50 (TS); 18 (veteran sub-sample)	History of criminal-legal involvement	X	X	
Fox et al. (2015)	US	Barriers to and facilitators of BUP post-release	Qualitative	2012	Transitions clinic providing MOUD; outpatient program providing treatment	Adult; < 5 yr incarceration; OUD; English- or Spanish-speaking	21	Jail; Prison; Release	X	X	
Frank et al. (2021)	US	Methadone motives and experiences	Qualitative	2014	Networks of community members	People who use drugs or receive methadone	42 (total patients and clinicians sample); not reported (patient sub-sample)	Court	X	X	
Gallagher et al. (2023)	US	Experiences of female drug court participants	Qualitative	NR	Drug court	Adult; female; drug court participant; spoke English; OUD	14	Court	X	X	X
Gryczynski et al. (2021)	US	Buprenorphine diversion during incarceration	Cross sectional study with qualitative	2019	Existing studies; intake to treatment program; outreach and referrals; community locations	Adult; past-yr OUD or opioid misuse; past 6 mo incarceration release	300	Jail; Prison	X	X	
Havnes et al. (2014)	Norway	Intentions and experiences with methadone and BUP use	Qualitative	NR	MOUD program at carceral facility	Adult; enrolled in MOUD program at the prison; consent; charged with offense while in program	12	Prison	X	X	
Hayashi et al. (2017)	Thailand	Barriers to methadone retention	Cross sectional study with qualitative	2011	Peer outreach (e.g., at clinics, on streets)	Adult; live in area; injected drugs < 6 mo	158 (SS); 16 (IS)	Prison		X	
Hoffman et al. (2023)	US	Barriers and facilitators to treatment and initial reintegration	Qualitative	2017	MOUD program/medical organization in community; clinician referrals	English-speaking; enrolled in opioid treatment program; incarceration history	42	Jail; Prison	X		
Hyatt et al. (2023)	US	Beliefs about NTX-XR compared to methadone	Cross sectional	2015	Treatment program during incarceration	Incarcerated; enrolled in AOD programming TC	1571 (TS); 1125 (substance use history sub-sample)	Jail; Prison		X	X
Kaplowitz et al. (2022a)	US	Attitudes about MOUD	Qualitative	2018	MOUD program at carceral facility	Adult; English-speaking; receiving MOUD in jail/prison program	40	Jail; Prison	X	X	
Kaplowitz et al. (2022b)	US	Preferences for MOUD	Qualitative	2018	MOUD program in community	Adult; OUD; receiving MOUD from program	40	Jail; Prison	X	X	X
Kaplowitz et al. (2023)	US	Factors that impact post-release treatment engagement	Qualitative	2018	MOUD program during incarceration	Adults; enrolled MOUD program	40	Jail; Prison; Release	X	X	
Komalasari et al. (2021)	Indonesia	Stigma relating to prison OAT; identify strategies to alleviate	Qualitative	2015	Medical program during incarceration	Incarcerated; > 6 mo methadone receipt OR current or < 6 mo injection drug use; no significant mental condition; not released before participation	57 (TS); 35 (incarcerated sub-sample)	Prison		X	
Larney et al. (2017)	Australia	Reasons to receive MOUD during incarceration and future intentions	Qualitative	2012	During incarceration	OUD history (MOUD receipt or documented diagnosis); incarcerated	46	Prison	X	X	
Liberman et al. (2021)	Kyrgyz Reupublic	Factors to consider when designing an OUD treatment decision aid	Qualitative	2016	Recently released via carceral facility	Adult; incarcerated; OUD via DSM-V; release date < 6 mo	36	Prison	X	X	
Liberman et al. (2022)	Kyrgyz Reupublic	Interest and uptake in methadone for pre-release people with OUD	Experimental study with survey and qualitative	2016	During incarceration	OUD; incarcerated; 8–180 days from release	125 (SS): 109 (received intervention), 16 (no intervention); 13 (IS)	Prison	X	X	
Makarenko et al. (2016)	Ukraine	Factors influencing willingness to initiate OAT	Cross sectional	2014	Community outreach sites	Adult; OUD; live or work in city; consent (including infectious disease testing)	1179 (TS); 424 (incarceration history sub-sample)	Prison	X		
Maradiaga et al. (2016)	US	If methadone challenges during incarceration affect subsequent attitudes	Qualitative	2012	Community-based organizations providing MOUD treatment	Adult; < 5 yr incarceration; OUD; English- or Spanish-speaking	21	Jail; Prison; Release		X	
Marshall et al. (2023)	Australia	Perspectives of OAT access in prison	Qualitative	2018	Existing study	Adult; injection drug use history; incarcerated > 3 mo; released from incarceration past yr; consent	48	Prison	X	X	
Martin et al. (2019)	US	Barriers to MOUD receipt during incarceration	Cross sectional	2017	In the community, upon release from incarceration	Adult; OUD via clinical criteria; received MOUD while incarcerated	214	Release; Community supervision, parole, or probation	X		
Matheson et al. (2022)	United Kingdom	Views on MOUD delivery options	Qualitative	2020	Social service agencies; community outreach; recovery communities	Homeless or at risk; experience with nonprescribed opioids or any MOUD	29 (TS); not reported (CL-involved sub-sample)	Court; Prison	X	X	
Michener et al. (2024)	US	Perceptions of post-release overdose risk	Qualitative	2021	At release from incarceration; service settings in community	Previously incarcerated in Massachusetts jail; received MOUD while incarcerated; living in community	38	Jail; Prison; Release	X		
Mukherjee et al. (2016)	Malaysia	Individual-level factors associated with interest for methadone in prison	Cross sectional	2014	During incarceration	Adult; opioid dependence diagnosis; incarcerated > 30 days; English- or Bahasa Malaysia-Speaking; consent	200	Prison		X	
Nguyen et al. (2024)	US	Barriers to accessing MOUD	Qualitative	2021	MOUD program/medical organization in community	Currently receiving OUD treatment; adult; English-speaking	30 (TS); not reported (CL-involved sub-sample)	Jail; Prison; Community supervision, parole, or probation	X	X	
O'Hara et al. (2022)	Moldova	Attitudes that shape uptake of carceral-delivered methadone	Cross sectional study with qualitative	2014	Medical organization in community	Adult; inject drugs; opioid dependence via ICD−10; released to community past 3 mo; access to methadone in prison and community	56 (SS); 44 (IS)	Prison		X	
Polonsky et al. (2016b)	Ukraine	Attitudes toward methadone; intentions to change substance use	Cross sectional	2010	During incarceration; upon release from incarceration	Living with HIV; adult; injected drugs < 30 days incarceration; currently incarcerated OR released	196 (TS); 99 (incarcerated sub-sample); 97 (release from incarceration sub-sample)	Prison; Release		X	
Polonsky et al. (2016a)	Moldova	Attitudes toward OAT	Cross sectional	2014	Community organization provides medical services	Adult; opioid dependence via ICD−10; released to communities < 3 mo; MOUD available in prison and community	56 (TS); 29 (OAT sub-sample)	Prison	X		
Russell et al. (2022a)	Canada	Experiences with OAT during incarceration; factors influencing outcomes	Cross sectional study with qualitative	2019	MOUD program at carceral facility	Incarcerated; OUD via clinic's requirement for MOUD; MOUD program for > 3 mo; statutory release or parole eligibility date scheduled < 6 mo; release location within catchment area; consent to baseline and follow-up	46	Prison	X	X	
Skogseth et al. (2024)	US	Factors facilitating MOUD engagement among women	Qualitative	2022	Social media; MOUD treatment programs in community	Adult; woman; consent; lifetime probation/parole/incarceration history OR currently incarcerated; reside in PA; lifetime history of MOUD program enrollment	42 (TS); 20 (patient sub-sample)	Jail; Prison; Community supervision, parole, or probation	X		
Stein et al. (2015)	US	Factors that influence detox patients	Cross sectional	2013	At intake to detox facility	Adult; opioid use; English-speaking; consent	485 (TS); 146 (CL-involved sub-sample)	Community supervision, parole, or probation; Pretrial release	X		
Stopka et al. (2024)	US	Perspectives and experiences of XR BUP	Qualitative	2021	At release from incarceration; service settings in community	Previously incarcerated in Massachusetts jail; received MOUD while incarcerated; living in community	38	Jail; Release		X	X
Swartz et al. (2022)	US	Influential facilitators and barriers on MOUD initiation	Qualitative	2019	Day center	Adult; English-speaking; experiences of homelessness; experiences of opioid overdose	29 (TS); 21 (CL-involved sub-sample)	Jail	X	X	
Thompson and Clegg (2023)	United Kingdom	Experiences of drug use offenders in and out of prisons	Cross sectional study with qualitative	NR	Unclear (mentioned a pharmacy)	Adult; record of offense; addicted to drugs; methadone program participant	5	Jail; Prison; Release		X	
Tomori et al. (2014)	Vietnam	Challenges and facilitators of reentry	Qualitative	2011	Outpatient HIV clinics; peer referrals	Male; inject drugs; released from "06 centers" in < 2 yrs	43	Compulsory drug detention centers		X	
Treitler et al. (2022)	US	Experiences and perspectives of MOUD during incarceration	Qualitative	2020	Re-entry treatment program	Adult; released from prison; OUD via self-report	53	Jail; Prison	X	X	X
Vail et al. (2021)	US	Attitudes and beliefs about MOUD continuation post-release	Qualitative	2018	MOUD program at carceral facility	Adult; English- or Spanish-speaking; currently receipt BUP; < 1 yr of release; consent to follow-up	22	Jail	X	X	
Velasquez et al. (2019)	US	Attitudes towards MOUD; re-entry treatment barriers and facilitators	Experimental study with qualitative	2016	Existing study; MOUD program/medical clinic	Adult; formerly incarcerated; released from jail in < 24 mo; enrolled in study or patient at BUP clinic; OUD	33	Release	X	X	X
Zelenev et al. (2018)	Ukraine	Barriers and willingness to participate in OAT	Cross sectional	2014	Treatment facilities	Never received MOUD; adult; opioid dependence via ICD−10	1613 (TS); 811 (sub-study); not reported (CL-involved sub-sample)	Jail; Prison; Police interaction	X		

^⁎^ =  all values in years, mean age unless otherwise specified

P/A =  preferences/attitudes; MOUD =  medications to treat opioid use disorder; NTX-XR =  naltrexone extended release; BUP =  buprenorphine; BUXR =  buprenorphine extended release; OAT =  opioid agonist treatment; HIV =  human immunodeficiency virus; OUD =  opioid use disorder; SD =  standard deviation; yr =  year; mo =  month; wk =  week; TS =  total sample; IS =  interview sub-sample; SS =  survey sub-sample; qualitative =  qualitative research such as interviews or focus groups; cross sectional =  cross sectional survey; release =  release from incarceration; NR =  not reported; X = yes; US =  United States

Caption: Each of the 44 articles included in this review is presented in the table above. For each included article, characteristics are described including location, aim, study design, year of data collection, setting, population, sample size criminal legal intercept, and which preference/attitudes were identified.

For each study, the reported participant characteristics varied, with most reporting age (81.8 %; 65.9 % reporting average) and gender or sex (93.2 %), while fewer reported race and/or ethnicity of participants (61.4 %). Studies often reported other patient characteristics, with over three-fourths reporting on current or past MOUD treatment use (75.0 %) or reporting on current or past incarceration histories (81.8 %). Reported sample characteristics varied by study design and study. The sample size ranged from 5 to 1613 participants. The average age was 38 years old (range: 31–44). Samples were 0.0–30.0 % female and 70.0–100.0 % male (sex), or 0.0–100.0 % women and 0.0–100.0 % men (gender). The categorization and reporting of race and/or ethnicity differed across studies (Appendix C). Regarding additional characteristics, participants often had incarceration histories (94.0 %) and MOUD treatment histories (70.0 %).

Study findings related to several different criminal-legal intercepts, though data were most often collected regarding incarceration experiences, in prison (75.0 %) and jail (44.7 %), followed by the period upon release from incarceration (25.0 %). Considerably fewer studies documented preferences/attitudes during community supervision (9.1 %), court (6.8 %), and the pre-arrest (police interactions) or arrest period (4.5 %). One study detailed experiences with child protective services, and another described participants who had unspecified criminal-legal involvement histories.

### 3.2 Stated preferences and attitudes toward MOUD

Across the 44 articles, most articles reported on positive (88.6 %, n = 39) or negative (86.4 %, n = 38) factors, with less than half reporting on mixed factors (45.5 %, n = 20). Often, within one study, multiple preferences/attitudes were expressed toward one or more MOUD types. Expression of preferences/attitudes toward MOUD as a whole (not specifying the type of medication) occurred most often (n = 31, 70.5 % of articles), expressed in equal measure positive (54.5 %) and negative (52.3 %). Three-fourths (n = 33) of studies reported preferences/attitudes on a specific MOUD type, either methadone, orally administered buprenorphine, injectable (extended-release) buprenorphine, or injectable (extended-release) naltrexone. Methadone was the most referenced MOUD type (68.2 %, n = 30), with slightly more articles referencing negative preference/attitudes than positive (59.1 % vs. 45.5 %). There were considerably fewer articles that referenced orally-administered buprenorphine (40.9 %, n = 18), with a balance of positive and negative (22.7 % each); and even fewer for buprenorphine or naltrexone injectables (n = 2 and n = 6, respectively). For the injectables, most articles reported positive (4.5 % buprenorphine and 13.6 % naltrexone) and some negative (4.5 % and 4.5 %). Of note, few patients across the 7 studies that documented injectable MOUD were receiving an injectable MOUD at the time of interview (n = 4, 0 %-33 % of patients were receiving an extended-release product; n = 3, receipt was unknown); many preferences/attitudes toward these products were hypothetical or, perhaps, reliant past experiences with injectable MOUD that were unclear in the articles. Overall, mixed valence factors were rare and, compared to other MOUD, methadone carried the most (25.0 %, n = 11 articles).

### 3.3 Influential social-ecological factors

Individual- and structural-level factors were present across most studies in this review, 93.2 %. Community-level factors appeared in 88.6 % of articles, and interpersonal factors in 59.1 %.

#### 3.3.1 Positive valence factors

##### 3.3.1.1 Individual factors

The most common individual-level factors to positively influence preferences/attitudes were beliefs about MOUD (n = 26, 59.1 % of articles) and intentions with MOUD (n = 21, 47.7 %), perceived MOUD effectiveness (e.g., ability of medication to help treat opioid issues or to work well generally) and, similarly, goals or outcomes one perceived to be associated with MOUD utilization (most often overdose risk, followed by avoiding reincarceration). Both beliefs and intentions for MOUD arose across all types, though most often when discussing an unspecified MOUD. Factors related to substance use also positively impacted preferences/attitudes; both continued or reduced substance use during treatment and abstinence intentions during treatment were referenced in 22.7 % of articles (n = 10). Additionally, 15.9 % of articles mentioned mental and physical health (e.g., pain management) and withdrawal avoidance as positive factors (n = 7), respectively. Other notable factors are demonstrated in Table 2.

**Table 2:** Count of factors shaping preferences for/positive attitudes toward medication to treat opioid use disorder (MOUD) across 44 studies.

**Theme**	**Category**	**Code**	**TOTAL**	**MOUD**	**MTD**	**BUP**	**BUP-XR**	**NTX-XR**
Individual	Mental and physical health		7	3	4			
	MOUD cognitive							
		Beliefs	26	13	3	5	1	4
		Knowledge	6	2	2			2
		Intentions	21	10	5	3	2	1
	MOUD physiologic response							
		Psychological	6	1	2	2		1
		Euphoria	4		1	2		1
		Side effects	1			1		
	Sense of self/personal disposition		6	5				1
	Demographics							
		Gender	2	1				1
	Stigma internalized		1				1	
	Substance use							
		Continue or reduce use	10	6	3			1
		Drug abstinence	10	3	3	2	1	1
	Withdrawal experiences and avoidance		7	3	2	2		
Interpersonal	Peer interactions or perceptions		5	1	2		1	1
	MOUD exposure via others		3	1	2			
	Social and familial support		8	5	3			
Community	Housing		3	3				
	Legal personnel interactions		1	1				
	Healthcare personnel interactions							
		Medical staff	2	2				
		Peer recovery specialist	2	2				
	Substance use availability and access		1	1				
	Treatment availability and access							
		Access to MOUD	8	4	3	1		
		Nonprescribed MOUD	3		1	2		
		Adjunctive services	3	3				
		Dosing administration	8	1	1	1	2	3
		Dosing take home	1	1				
		MOUD program rules	14	5	4	3	1	1
		Social/political in clinic	3	1	2			
	Treatment quality							
		Sufficient dose	1	1				
		Tailored program	2	1	1			
		Interruptions	2	1			1	
Structural	Economic conditions							
		Costs	2	1	1			
		Employment	2	2				
	Legal policies							
		Correctional administration distrust or fear	2	2				
		Legal pressure	1		1			
	Criminal-legal systems involvement							
		Dependent on criminal-legal intercept	6	4	1		1	
		Incarceration history	2	2				
		Life responsibilities	4	2			1	1
		Planning post-release	5	3	1			1
		Planning post-release (facilitated)	3	3				
	Stigmas and related ideologies							
		Drug criminalization	5	1	3	1		
		Drug free ideology	1			1		
		Stigma: criminal record	1		1			
		Stigma: methadone	3			3		
		Stigma: MOUD	4	1	1	1	1	
		Stigma: substance use	1		1			
	Substance use supply		1	1				

TOTAL =  total number of articles containing data on each code; MOUD =  medications to treat opioid use disorder; MTD =  methadone; NTX-XR =  naltrexone extended release; BUP =  buprenorphine orally administered; BUP-XR =  buprenorphine extended release; CPS =  child protective services

Caption: This table includes each social-ecological factor that was identified across all articles in this review to positively impact preferences/attitudes toward medications to treat opioid use disorder. Each factor includes a count to represent the number of papers where the factor is present, and each factor is also identified within the social-ecological level.

##### 3.3.1.2 Interpersonal factors

Fewer factors of positive valence were identified at the interpersonal level. The most common facilitators/factors positively associated with MOUD preferences/attitudes were social and family support (n = 8, 18.2 %) and peer interactions or perceptions (n = 5, 11.4 %). Family relationships were positive, influential factors in relation to MOUD unspecified and methadone, while peer interactions were additionally mentioned in relation to the injectable MOUD types. For example, injectable MOUD is favorable because it leads to fewer peer altercations.

##### 3.3.1.3 Community factors

The most common positively influential factor at the community-level across studies was MOUD program rules (n = 14, 31.8 %) divided among the periods of during incarceration (e.g., non-daily dosing for some medications, facilitated receipt upon release, satisfaction with dose and intake processes, provided change in social setting) and in the community (e.g., likes daily routine, promise of fewer clinic visits). This spanned all types of MOUD. Of similar frequency was the administration of MOUD (n = 8, 18.2 %), such as the frequency and route of dosing (i.e., number of times attend clinic, long-acting nature of medication), which was most often attributed to injectable formulations. MOUD access factors were also referenced as important (n = 8, 18.2 %), such as general access (e.g., ability to receive MOUD in a given setting) and the variety of available MOUD options at a given setting.

##### 3.3.1.4 Structural factors

A variety of structural factors were commonly found across articles, often related to the criminal-legal system and stigma. A given criminal-legal intercept often positively impacted an individual’s perception of MOUD utilization (e.g., electing to receive a MOUD during the post-release period due to known overdose risk) (n = 6, 13.6 %), as individuals referenced planning for the post-release period (n = 5, 11.4 %) and others. Management of other life responsibilities was also strongly valued when describing perceptions of MOUD receipt and one’s criminal-legal intercept (n = 4, 9.1 %). For example, when desiring child reunification post-release, a participant may have felt positively about receiving MOUD. Stigmas that positively impacted MOUD perceptions were present across many domains of identity, including toward methadone (n = 3, 6.8 %) and MOUD as a whole (n = 4, 9.1 %). These stigmas may have influenced an individual’s desire to receive MOUD or a specific type of MOUD; most commonly, stigma toward methadone was mentioned exclusively in conversations about positive perceptions of orally administered buprenorphine. For example, the relative lack of stigma associated with buprenorphine, in contrast to methadone. Drug criminalization (n = 5, 11.4 %) also arose in this category, demonstrating how individuals referenced the use of MOUD as a tool to avoid a return to use and subsequent involvement with the legal system (or incarceration).

#### 3.3.2 Negative valence factors

##### 3.3.2.1 Individual factors

Across the articles in this review, negative attitudes toward/preferences against MOUD(s) were present within all social-ecological levels. At the individual level, anticipated or experienced side effects from MOUD were the most common negative factor (n = 15, 34.1 % of articles). These side effects varied and were present across most MOUD types (e.g., unintended side effects, nausea, bone decay, injection site pain). Other influential individual-level factors included a desire to avoid all withdrawal (n = 10, 22.7 %), resulting in a desire to avoid MOUD, often for methadone or a buprenorphine formulation. Additionally, common influential beliefs (n = 13, 29.5 %) included distrust or uncertainty towards a MOUD’s pharmacology (e.g., properties of the medication such as lack of control over dose or presence of medication in body) and disbelief in the MOUD’s effectiveness (across methadone and buprenorphine products). Factors related to substance use also contributed to attitudes/preferences, such that continued or reduced use of substances (n = 5, 11.4 %) deterred patients from MOUD, especially orally administered buprenorphine; and desire for abstinence (n = 6, 13.6 %) deterred them from methadone, specifically.

##### 3.3.2.2 Interpersonal factors

Peers were the most common interpersonal factor to negatively influence attitudes of MOUD (n = 8, 18.2 %) for MOUD, except for the injectables. For example, loss of peer groups or deterrence from peers who receive MOUD due to stigma. Table 3 describes additional influential factors.

**Table 3:** Count of identified factors shaping negative preferences/attitudes toward medication to treat opioid use disorder (MOUD) by type.

**Theme**	**Category**	**Code**	**TOTAL**	**MOUD**	**MTD**	**BUP**	**BUP-XR**	**NTX-XR**
Individual	Mental and physical health		2		1	1		
	MOUD cognitive							
		Beliefs	13		9	2	2	
		Knowledge	4	2	2			
		Intentions	5	2	3			
	MOUD physiologic response							
		Psychological	1		1			
		Euphoria	1		1			
		Side effects	15	5	6	3	1	
	Sense of self/personal disposition		4		3	1		
	Demographics							
		Age	1		1			
		Gender	1	1				
		Race	1					1
	Stigma internalized		2	1	1			
	Substance use							
		Continue or reduce use	5	3		2		
		Drug abstinence	6		6			
	Withdrawal experiences and avoidance		10	3	5	1	1	
Interpersonal	Peer interactions or perceptions		8	3	4	1		
	Social and familial support		2	1	1			
Community	Housing		6	6				
	Legal personnel interactions		10	5	5			
	Healthcare personnel interactions							
		Medical staff	3	3				
	Social service programs		2	2				
	Substance use availability and access		1	1				
	Transportation		5	4	1			
	Treatment availability and access							
		Access to MOUD	4	3		1		
		Nonprescribed MOUD	2			1	1	
		Adjunctive services	1	1				
		Dosing administration	5		1	2	1	1
		Dose protocol	3	1	1	1		
		MOUD program rules	24	8	12	3	1	
		Social/political in clinic	6	2	4			
	Treatment quality							
		Incarceration as detox	2	1	1			
		Treatment history during incarceration	9	3	5	1		
		Sufficient dose	2	1		1		
		Tailored program	3	1	2			
		Interruptions	4	2	1	1		
Structural	Economic conditions							
		Costs	2	1			1	
		Employment	2	2				
	Insurance		3	3				
	Legal policies							
		Correctional administration distrust or fear	4	3	1			
		Legal pressure	2	1	1			
		Documentation needs	2	2				
		Parole probation conditions	2	2				
	Criminal-legal systems involvement							
		Dependent on criminal-legal intercept	6	3	2	1		
		Legal involvement (unspecified)	1	1				
		Incarceration history	1	1				
		Life responsibilities	4	3	1			
		Planning post-release	2	1	1			
		Planning post-release (facilitated)	1	1				
	Stigmas and related ideologies							
		Drug free ideology	14	5	8	1		
		MOUD criminalization	1		1			
		Stigma: criminal record	2	1	1			
		Stigma: methadone	11		10	1		
		Stigma: MOUD	11	9		2		
		Stigma: substance use	1		1			

# =  total number of references across articles; MOUD =  medications to treat opioid use disorder; MTD =  methadone; NTX-XR =  naltrexone extended release; BUP =  buprenorphine orally administered; BUP-XR =  buprenorphine extended release; CPS =  child protective services

Caption: This table includes each social-ecological factor that was identified across all articles in this review to negatively impact preferences/attitudes toward medications to treat opioid use disorder. Each factor includes a count to represent the number of papers where the factor is present, and each factor is also identified within the social-ecological level.

##### 3.3.2.3 Community factors

MOUD program rules during incarceration and in the community were referenced as negative influences on MOUD preferences/attitudes in over half of the studies (n = 24, 54.5 %). For example, participants referenced the weight of daily/frequent clinic attendance, regular urine toxicology screens, feelings of extreme restriction, and complex admission requirements (community); and interruptions in treatment at intake to incarceration, visibility of medication receipt to peers and lack of confidentiality, rapid taper, leniency in MOUD provision to others, and transfer to facilities or communities without MOUD access (incarceration). An individual’s prior negative experiences during incarceration were specifically noted (n = 9, 20.5 %), including detoxing from nonprescribed opioids or MOUD during incarceration, which adversely impacted future desire for MOUD. These attitudes were often associated with methadone or unspecified MOUD. Additional diverse factors negatively influenced MOUD preferences/attitudes: interactions with criminal-legal personnel (n = 10, 22.7 %), housing (lack of and instability) (n = 6, 13.6 %), social or political climate of the treatment facility (n = 6, 13.6 %; e.g., harassment or violence when entering treatment clinic), transportation (lack of and unreliability) (n = 5, 11.4 %), and dosing administration process (n = 5, 11.4 %; e.g., dislike for oral buprenorphine taste, injectable MOUD needles).

##### 3.3.2.4 Structural factors

Structurally, negative attitudes toward MOUD were largely shaped by stigmas and ideologies, including one that individuals must/should find recovery without any substances (including medications) (n = 14, 31.8 %; over half methadone) and acknowledgment of stigma or holding stigma toward methadone (e.g., methadone’s association with other feared diseases or undesirable social qualities) (n = 11, 25.0 %) or MOUD as a whole (n = 11, 25.0 %). These stigmas were not present for injectable MOUD. In addition to stigmas and ideologies, given criminal-legal intercepts also exerted a negative influence on MOUD preferences/attitudes (n = 6, 13.6 %), such as the need to address basic needs before MOUD during the post-release period and generally lower endorsement of methadone during incarceration (vs. post-release) or different intentions with methadone during incarceration (vs. in the community).

#### 3.3.3 Mixed valence factors

Influential factors of mixed valence or non-positive motivators (i.e., identified as a stimulus for receipt, though came with tension) were less represented in the data. At the individual-level these factors included: beliefs about medication effectiveness or pharmacological properties (e.g., perceived no difference between orally administered or injected) (n = 6, 13.6 %), intentions or desire for MOUD to meet goals related to overdose risk or reincarceration (i.e., sought MOUD only to reduce overdose risk at post-incarceration or to avoid law enforcement and reincarceration) (n = 5, 11.4 %), and continued or reduced substance use while receiving MOUD (e.g., external factors motivated treatment, found MOUD acceptable but waited until positive urine toxicology to enter treatment) (n = 4, 9.1 %). At the community- and structural-levels, MOUD program rules (n = 3, 6.8 %) and moving through criminal-legal intercepts or appraising oneself during intercepts (n = 3, 6.8 %) were influential (e.g., desire to receive MOUD while incarcerated then taper toward the end of parole period, desire to receive MOUD post-release not during incarceration) (Appendix E).

## 4 Discussion

Across the 44 studies in this scoping review, we identified a range of diverse factors across numerous countries that shaped preferences for and attitudes towards MOUD, often related to MOUD broadly and methadone, specifically. Fewer studies detailed preferences/attitudes toward orally administered buprenorphine, and considerably fewer referenced injectables. Most factors were concentrated at the individual, community, and structural levels of experiences during incarceration in jail or prison or the immediate post-release period. Preferences and attitudes appeared to be inexplicably informed by and intertwined with structural barriers and facilitators, such that when sharing their preferences for and attitudes toward MOUD, patients often described how they were barred from accessing MOUD or how systems supported the facilitation of MOUD, therefore, leading to the formation of their intentions and decisions. Holistically, findings were consistent with established literature on healthcare preferences (Brennan and Strombom, 1998). Additionally, this scoping review extends literature on social-ecological factors that shape opioid use and public health responses to opioids (Jalali et al., 2020, Russell et al., 2022b), by highlighting factors that are unique to the criminal-legal system and, therefore, critical to the health of people with OUD who are often involved with this system (Wakeman, 2017). Notable criminal-legal-specific social-ecological factors include: treatment access during incarceration, treatment quality, including negative experiences during prior incarcerations (i.e., received substandard treatment or forcibly detoxed) (community-level), and a given criminal-legal intercept (e.g., wanting to receive methadone during the post-release period specifically to reduce overdose risk) (structural-level). Identification of these important factors illuminates how MOUD preferences/attitudes are informed by one’s criminal-legal status.

Given that different factors impact MOUD preferences/attitudes and utilization between differing periods of criminal-legal involvement, it is especially critical to engage in further research on patient preferences during understudied intercepts like the pre-arrest period and court. These individuals often face unique community and structural barriers to treatment, as documented by clinicians and officers (e.g., community supervision conditions) (Booty et al., 2023, Kang et al., 2024). More data on MOUD preferences/attitudes during these intercepts could support the development, implementation, and operations of public health interventions that aim to initiate treatment earlier in the criminal-legal cascade (e.g., deflection (National Conference of State Legislatures, 2023)) and retain individuals later in the cascade (e.g., programming offered via community supervision). The improvement of MOUD delivery for these individuals could increase treatment engagement and enhance other health outcomes.

Several social-ecological factors, previously shown to shape healthcare decisions and health outcomes among people with OUD involved in the criminal-legal system (Krawczyk et al., 2024, Martin et al., 2023, Scott et al., 2021), were underreported by studies in this review. For example, sociodemographic characteristics like race, ethnicity, gender, and age were infrequently mentioned as being influential to MOUD preferences/attitudes. This finding aligns with prior reviews on MOUD perceptions and experiences in the general population with OUD (Cioe et al., 2020, Flam-Ross and Zampini, 2025). Some preliminary evidence suggests that, of those receiving MOUD during incarceration, preferences for MOUD type do not differ by racial and ethnic groups; however, preferences for MOUD type *initiated* during incarceration differ between non-White and White individuals (Berk et al., 2025a). This suggests that there may be an interplay between criminal-legal involvement, key demographic characteristics, and preferences. Future research could assess multi-level factors that shape MOUD decision-making processes during incarceration by these demographic characteristics. Also understudied in previous original research and worthy of future investigation are the social and political determinants of health, like the substance use supply, non-opioid substance use, socio-economic factors (e.g., income), housing and systemic supports for housing, and MOUD dose (Bolshakova et al., 2024, Chambers et al., 2023, Sugarman et al., 2020).

By MOUD type, there were differences in the frequency and valence of influential factors on preferences. Methadone was referenced in more articles (i.e., n = 30; 68.2 %), in contrast to other types, presumably because it is an older, more widely available in community settings, and more established globally (Dydyk et al., 2025). Negative preferences/attitudes toward methadone were commonly expressed, more than toward other MOUD, and were persistent across all social-ecological levels (e.g., desire to avoid withdrawal from methadone, prior negative experiences during incarceration). Notably, structural-level ideologies, such as stigma toward MOUD, were particularly negative toward methadone and influential on participants’ preferences/attitudes. Indeed, bias against and stigma toward methadone are pervasive, proliferate throughout social support networks, and often shape both patient and clinician perspectives on treatment options and harms, even in contrast to other MOUD (Crapanzano et al., 2019, Earnshaw, 2020, Madden et al., 2021, Meyer et al., 2025). Stigma impacts patient decisions and health outcomes (i.e., deterrent to initiation or continuation) and often comes with mistrust toward clinics and medical systems in general (Randall-Kosich et al., 2020, Saunders et al., 2020, Woo et al., 2017). Some strategies could reduce the harms of stigma toward MOUD, such as practices to foster trust between patients and medication via providers, counselors, peer support, and familial networks (Earnshaw, 2020, Saunders et al., 2020).

Perceived or experienced side effects or adverse effects were a common reason to dislike a given medication and elect to favor or receive an alternative MOUD. In this review, concerns about side effects and adverse reactions were expressed about nearly all MOUD, with methadone most frequently associated with both side effects and fear of withdrawal, factors that negatively influenced preferences for its use. Research finds that there is limited evidence demonstrating differences in risk of adverse effects by MOUD type, and, of the studies that exist, there is a high risk of bias (Meyer et al., 2025). Enhancing patient knowledge about MOUD types is important, as a prior review of literature found that knowledge may be a strong indicator of MOUD choice (Cioe et al., 2020), whereby misinformation or lack of information can exert considerable force on treatment decision-making. To understand experiences and effectively manage side and adverse effects, clinical staff or providers can consult patients: discuss side effects, and determine preference-congruent and appropriate MOUD type, timing of administration, and dose (Meyer et al., 2025). Other prominent individual-level factors from this review, like negative beliefs about MOUD effectiveness, could be potentially modified in such a clinical interaction, with the intention to enhance MOUD knowledge generally, clarify treatment preferences, and identify ideal individualized treatment pathways (Dydyk et al., 2025). Lastly, given the prevalence of side or adverse effects as determinants of MOUD decisions, it may be prudent to systematically investigate these effects at a larger scale and perhaps gain clarity on additional factors that influence negative side effects (e.g., dose, administration, medication and patient characteristics).

Perceptions of long-acting injectables (buprenorphine and naltrexone) were mostly positive from participants in the included studies. However, most had not received an injectable medication at the time of the interview. Participants anticipated multilevel advantages of injectables, including beliefs about effectiveness, familial support of the MOUD, and superior route of administration and routine of dosing; consistent with general populations with OUD (Flam-Ross and Zampini, 2025, Saunders et al., 2020). As Flam-Ross and Zampini (2025) found in a review of literature, injectable buprenorphine may be particularly attractive in a social environment laden with stigma toward MOUD because the pharmacology of the medication, regulations that govern receipt (e.g., less clinic, less missing work), and patient-experienced outcomes (e.g., reductions in use and visibility) of long-term injectable use are a conduit to “normalcy” for individuals. Given promising outcomes, injectables should be provided as an *option* and not the only MOUD (Martin et al., 2022). Indeed, for some aspects of the injectable may outweigh the benefits, such as the negatives identified in this review (i.e., concern about injectable-specific properties like inability to control the daily medication dose). As injectable MOUD diffuses throughout treatment systems, knowledge of and experiences with the medications will increase for patients and staff (Flam-Ross and Zampini, 2025). Enhancing both *evidence-based* knowledge and accessibility (e.g., costs) of injectable MOUD will likely support patient autonomy for informed decision-making with medical providers. Additionally, since most studies in the present review collected data in 2018, it is prudent to re-collect data on perceptions of injectables from the patient population.

The community and structural-level factors identified in this review may be the most relevant for organizational- and systems-level change to better meet the MOUD treatment preferences of individuals with OUD across the criminal-legal system. Many of these factors are well-established in the literature (National Academies of Sciences, 2019), though they are worth underscoring. Daily or near-daily dosing, lack of MOUD options available, and requirements that delayed or prohibited dosing were seen as deterrents for many participants, consistent with prior research with the general population with OUD (Lowry et al., 2024). During periods of incarceration, patients uniquely described how being held without treatment, without timely treatment, or with inadequate treatment (e.g., low dose, rapid taper) uniquely shaped future decisions for MOUD, either in favor or against MOUD or a given type. Therefore, the absence of low-threshold and accessible MOUD further contributes to the harmful force of incarceration, an experience that is already damaging to individuals’ well-being (Vanjani, 2017). Given how critical it is for patients to receive MOUD during incarceration for their health and treatment outcomes (Berk et al., 2025b, Cates and Brown, 2023), there is a pressing need for all jails and prisons to navigate MOUD provision barriers (e.g., stigma, workforce issues (Grella et al., 2020)). Collectively, these findings highlight how MOUD clinic practices impact patient preferences for this life-saving treatment and that points of contact, such as incarceration entry and community re-entry, should be optimized for low-threshold initiation/continuation and support (e.g., decisional support, re-entry plans).

### 4.1 Limitations and strengths

There are limitations to this scoping review. First, articles included in the scoping review only include published peer reviewed articles and, therefore, exclude potential insights from the gray literature, dissertations, and articles yet to be published by the search date. The decision to include only published articles intentionally excludes products that have not met the rigor of peer review (Adams et al., 2016). Second, six peer reviewed databases were searched for this review to cover a breadth of journals from different disciplines; however, additional published literature may exist outside of these databases or outside of the English language. Third, the screening inclusion criteria for this study required that the authors report the relevant concepts (MOUD, criminal-legal involvement, preferences/attitudes) within the abstract of the article. Therefore, studies on this topic that exclude one of these concepts from their abstract were omitted from the review; presenting an area for future research. Fourth, the distillation and interpretation of data using content analysis may limit the richness of data presented in each article, which is certainly a limitation, though a viable method to condense this quantity of text. Fifth, the data collection dates for included studies (most often 2018) limit inferences about patients' *most current* experiences with MOUD. Updated data on these topics might reveal different patient MOUD preferences due to recent structural changes that alter access to and experiences receiving MOUD (e.g., enhanced flexibilities through policy changes). Despite these limitations, this scoping review provides valuable insight into treatment preferences for individuals with OUD who are at a heightened risk for overdose and related mortality. Synthesized data from this review presents an opportunity to understand and respond to treatment needs among this population during the evolving overdose crisis.

### 4.2 Conclusions

This scoping review synthesized contemporary research findings to illuminate the complex factors influencing patient preferences for and attitudes toward MOUD across carceral and post-release settings. These preferences were shaped by a dynamic interplay of individual beliefs, interpersonal relationships, community and clinic conditions, and structural factors, with experiences during incarceration emerging as especially formative. While preferences are often treated as personal or cognitive decisions, this review highlights how they are fundamentally shaped by access, availability, institutional practices, and deeply embedded stigma - especially toward methadone. Community and structural determinants, such as clinic logistics (e.g., dosing protocols) and incarceration-related constraints (e.g., differential rules, lack of treatment), not only limit the autonomy of people with OUD but also have long-term implications for MOUD engagement. Importantly, the review also identified the promise of long-acting injectables, which were often met with more favorable perceptions, albeit based more on expectations than experience. Despite decades of research, gaps remain in understanding how individual (e.g., sociodemographic) and structural (e.g., policy changes) factors shape MOUD decision-making, particularly within underexplored criminal-legal contexts. As MOUD policy and practice continue to evolve, future research must prioritize input from patients to ensure that MOUD delivery aligns with the preferences and needs of those receiving treatment.

## Glossary

Medications to treat opioid use disorder (MOUD) includes the treatments of methadone, buprenorphine, and naltrexone; Opioid use disorder (OUD) is a chronic medication condition characterized by dependence on the use of opioids that causes interference with daily life activities. PRISMA stands for the Preferred Reporting Items for Systematic Review and Meta-Analysis, while PRISMA-ScR is the PRISMA Extension for Scoping Reviews.

## Ethics approval and consent to participate

Not applicable.

## Funding

This research was supported by the National Institute on Drug Abuse (NIDA) by the National Institutes of Health (NIH) [PI Bailey 1F31DA061612–01; PI Martin, U01-DA050442–01]. The content is solely the responsibility of the authors and does not necessarily represent the official views of the NIH. The NIH had no role in study design, administration, interpretation of findings, or dissemination of the study.

## CRediT authorship contribution statement

**Rosemarie A. Martin:** Writing – review & editing, Funding acquisition, Conceptualization. **Claudia Stagoff-Belfort:** Writing – review & editing, Validation, Formal analysis. **Shira I. Dunsiger:** Writing – review & editing, Conceptualization. **Amelia Bailey:** Writing – original draft, Funding acquisition, Formal analysis, Conceptualization. **Jaclyn M.W. Hughto:** Writing – review & editing, Conceptualization.

## Declaration of Competing interest

The authors declare that they have no known competing financial interests or personal relationships that could have appeared to influence the work reported in this paper.

## Figures and Tables

**Table:** 

**SECTION**	**ITEM**	**PRISMA-ScR CHECKLIST ITEM**	**REPORTED ON PAGE #**
**TITLE**			
Title	1	Identify the report as a scoping review.	1
**ABSTRACT**			
Structured summary	2	Provide a structured summary that includes (as applicable): background, objectives, eligibility criteria, sources of evidence, charting methods, results, and conclusions that relate to the review questions and objectives.	2
**INTRODUCTION**			
Rationale	3	Describe the rationale for the review in the context of what is already known. Explain why the review questions/objectives lend themselves to a scoping review approach.	4
Objectives	4	Provide an explicit statement of the questions and objectives being addressed with reference to their key elements (e.g., population or participants, concepts, and context) or other relevant key elements used to conceptualize the review questions and/or objectives.	4
**METHODS**			
Protocol and registration	5	Indicate whether a review protocol exists; state if and where it can be accessed (e.g., a Web address); and if available, provide registration information, including the registration number.	4
Eligibility criteria	6	Specify characteristics of the sources of evidence used as eligibility criteria (e.g., years considered, language, and publication status), and provide a rationale.	5
Information sources⁎	7	Describe all information sources in the search (e.g., databases with dates of coverage and contact with authors to identify additional sources), as well as the date the most recent search was executed.	5
Search	8	Present the full electronic search strategy for at least 1 database, including any limits used, such that it could be repeated.	5
Selection of sources of evidence†	9	State the process for selecting sources of evidence (i.e., screening and eligibility) included in the scoping review.	5
Data charting process‡	10	Describe the methods of charting data from the included sources of evidence (e.g., calibrated forms or forms that have been tested by the team before their use, and whether data charting was done independently or in duplicate) and any processes for obtaining and confirming data from investigators.	6
Data items	11	List and define all variables for which data were sought and any assumptions and simplifications made.	6
Critical appraisal of individual sources of evidence§	12	If done, provide a rationale for conducting a critical appraisal of included sources of evidence; describe the methods used and how this information was used in any data synthesis (if appropriate).	n/a
Synthesis of results	13	Describe the methods of handling and summarizing the data that were charted.	6
**RESULTS**			
Selection of sources of evidence	14	Give numbers of sources of evidence screened, assessed for eligibility, and included in the review, with reasons for exclusions at each stage, ideally using a flow diagram.	6, Fig. 1
Characteristics of sources of evidence	15	For each source of evidence, present characteristics for which data were charted and provide the citations.	6, Table 1
Critical appraisal within sources of evidence	16	If done, present data on critical appraisal of included sources of evidence (see item 12).	n/a
Results of individual sources of evidence	17	For each included source of evidence, present the relevant data that were charted that relate to the review questions and objectives.	6–9, Table 1
Synthesis of results	18	Summarize and/or present the charting results as they relate to the review questions and objectives.	6–9, Table 2, Table 3
**DISCUSSION**			
Summary of evidence	19	Summarize the main results (including an overview of concepts, themes, and types of evidence available), link to the review questions and objectives, and consider the relevance to key groups.	9–10
Limitations	20	Discuss the limitations of the scoping review process.	12
Conclusions	21	Provide a general interpretation of the results with respect to the review questions and objectives, as well as potential implications and/or next steps.	12–13
**FUNDING**			
Funding	22	Describe sources of funding for the included sources of evidence, as well as sources of funding for the scoping review. Describe the role of the funders of the scoping review.	14, Appendix C

**Table:** 

	***MeSH and Keywords***	**# results**
Medications to treat opioid use disorder	("Opiate Substitution Treatment"[Mesh] OR "Opioid-Related Disorders/drug therapy"[Mesh]) OR ((Opiate Substitution Treatments[Title/Abstract] OR Opiate Replacement Therapy[Title/Abstract] OR Opioid Replacement Therapy[Title/Abstract] OR Opioid Substitution Therapy[Title/Abstract] OR Opioid Substitution Treatment[Title/Abstract] OR Opioid Substitution Treatments[Title/Abstract] Opioid Maintenance Treatment[Title/Abstract] OR Opioid Maintenance Treatments[Title/Abstract] OR Medication Assisted Treatment[Title/Abstract]) OR (buprenorphine[Title/Abstract] OR methadone[Title/Abstract] OR naltrexone[Title/Abstract]) OR (medications to treat opioid use disorder[Title/Abstract] OR medications for opioid use disorder[Title/Abstract]) OR (Opioid Dependence[Title/Abstract] OR Opioid Dependences[Title/Abstract] Opioid Addiction[Title/Abstract] OR Opiate Dependence[Title/Abstract] OR Opiate Addiction[Title/Abstract] OR Opioid Abuse[Title/Abstract] OR Opiate Abuse[Title/Abstract] OR Opioid Misuse[Title/Abstract] OR Opioid Use Disorder[Title/Abstract] OR Opioid Use Disorders[Title/Abstract] OR Prescription Opioid Abuse[Title/Abstract] OR Prescription Opioid Misuse[Title/Abstract]) AND (medicat⁎[Title/Abstract]))	16672
Preferences	((Patient Preferences[Title/Abstract]) OR (attitude⁎[Title/Abstract] OR preference⁎[Title/Abstract] OR perspective⁎[Title/Abstract] OR interest⁎[Title/Abstract] OR intent⁎[Title/Abstract] OR decision⁎[Title/Abstract] OR choice[Title/Abstract] OR choose[Title/Abstract] OR chose[Title/Abstract] OR desir⁎[Title/Abstract] OR select⁎[Title/Abstract] OR endorse⁎[Title/Abstract] OR opinion⁎[Title/Abstract])) OR ("Attitude" [Mesh] OR "Patient Preference"[Mesh])	5630832
Individuals with criminal-legal histories	((Incarcerations[Title/Abstract] OR Imprisonment[Title/Abstract] OR Imprisonments[Title/Abstract] OR Criminal[Title/Abstract] OR Offenders[Title/Abstract] OR Offender[Title/Abstract]) OR (incarcerat⁎[Title/Abstract] OR criminal⁎[Title/Abstract] OR offender⁎[Title/Abstract] OR misdemeanor⁎[Title/Abstract] OR felon⁎[Title/Abstract] OR prison⁎[Title/Abstract] OR jail⁎[Title/Abstract] OR parole[Title/Abstract] OR probat⁎[Title/Abstract] OR inmate⁎[Title/Abstract] OR detention[Title/Abstract] OR convict⁎[Title/Abstract] OR correction⁎[Title/Abstract] OR penitentiary[Title/Abstract] OR discharge⁎[Title/Abstract] OR release⁎[Title/Abstract] OR community supervision[Title/Abstract] OR under supervision[Title/Abstract] OR arrest⁎[Title/Abstract] OR pre-arrest[Title/Abstract] OR violation⁎[Title/Abstract] OR infraction⁎[Title/Abstract] OR court⁎[Title/Abstract])) OR ("Incarceration"[Mesh] OR "Criminals"[Mesh])	1837393
	#1 AND #2 AND #3	929
	Since 2014	742

**Table:** 

	**N or mean**	**% or SD**
**Article year (mode, range)**	2022	2014–2024
**Conflicts of interest (reported)**	35	79.5
Yes	5	11.4
**Funding for study (reported)**	41	91.1
Any funding	37	84.1
*Private sector*	3	6.8
*Government or non-profit*	34	77.3
**Country**		
United States	24	54.6
Moldova	3	6.8
Ukraine	3	6.8
Australia	2	4.6
Indonesia	2	4.6
Kyrgyz Republic	2	4.6
United Kingdom	2	4.6
Canada	1	2.3
France	1	2.3
Malaysia	1	2.3
Norway	1	2.3
Thailand	1	2.3
Vietnam	1	2.3
**Study design**		
Qualitative study	26	59.1
Cross-sectional survey	9	20.5
Cross sectional survey plus qualitative interviews	6	13.6
Experimental study with survey and/or qualitative interviews	3	6.8
**First year of data collection (mode, range)**	2018	2010–2022
**Focus of journal where published**		
Addiction or substance use	32	72.7
Criminal legal	3	6.8
Other health or healthcare	9	20.5
**Study sample, data provided on:**		
Age	36	81.8
Gender or sex	41	93.2
*Gender*	14	31.8
*Sex*	7	15.9
*Gender or sex, unclear*	20	45.5
Race and/or ethnicity	27	61.4
*Race*	5	11.4
*Ethnicity*	3	6.8
*Race and ethnicity*	5	11.4
*Race/ethnicity, combined*	14	31.8
Current or past treatment use	33	75.0
Current or past incarceration	36	81.8
Preferences for MOUD		
*MOUD, unspecified*	39	88.6
*MOUD, specified type*	31	70.5
*MOUD, specified injectable*	7	15.9
**Criminal-legal intercept related to results:**		
Pre-arrest	1	2.3
Arrest	1	2.3
Court	3	6.8
Jail	21	47.7
Prison	33	75.0
Release from incarceration	11	25.0
Community supervision	4	9.1
*Child protective services*	1	2.7
*Unspecified criminal-legal involvement*	1	2.7

CL =  criminal-legal; MOUD =  medications to treat opioid use disorder; SD =  standard deviation

**Table:** 

**Theme**	**Category & Code Names**	**Definition**: “Discussion of…”	**Example:**	**Example articles:⁎**

Individual	**Mental and physical health**	Medical issues or pain management issues, potentially related to OUD, that inform MOUD preferences/attitudes.	MOUD helps manage chronic pain, methadone noted to facilitate pain management, buprenorphine less so. Limit disease spread via injection substance use (e.g., HIV, HCV, other infections), decrease medical and behavioral incidents, other medical needs.	Liberman et al. (2021); Treitler et al. (2022)
	**MOUD cognitive**	MOUD cognitions and how they inform preferences/attitudes.		
	Beliefs	Beliefs of MOUD and how it informs preferences/attitudes, spanning MOUD dependency, MOUD effectiveness, and perceptions of pharmacology.	MOUD to address addiction (minimize cravings, stabilizing, reduce substance use, avoid triggers, want “normal life,” regulate), MOUD helps or does not help an individual, MOUD benefits others.	Culbert et al. (2015); Hyatt et al. (2023)
	Knowledge	Knowledge of MOUD and how it informs preferences/attitudes, spanning a lack of knowledge or awareness for MOUD, examples of knowledge of MOUD, and myths about MOUD.	Unaware of MOUD options, desire to learn more, greater knowledge associated with positive attitudes.	Mukherjee et al. (2016); Stopka et al. (2024)
	Intentions	Intentions with MOUD utilization, spanning continued/stopping receipt, goals associated with MOUD, interest in initiation, and MOUD’s role in recovery.	Goals of MOUD utilization around avoiding reincarceration, goal setting or achieving goals, overdose risk.	Hoffman et al. (2023); (Russell et al., 2022a)
	**MOUD physiologic response**	Any physiologic response to MOUD utilization, either experienced or imagined.		
	Psychological	Psychological response to MOUD utilization, either experienced or imagined.	Enhanced sense of control, feeling more calm, feelings of stability, preference for autonomy.	Liberman et al. (2021); Treitler et al. (2022)
	Euphoria	Psychological response to MOUD utilization, specifically feelings of euphoria or “being high” either experienced or imagined.	Preference for MOUD types for inducing feelings of euphoria or for not inducting feelings, for the latter often buprenorphine (after stabilization) and naltrexone perceived to not cause euphoric feelings.	Gryczynski et al. (2021); O'Hara et al. (2022)
	Side effects	Side effects in response to MOUD utilization, either experienced or imagined.	Physical effects (general effects, bone aches, pain, headaches, nausea, constipation, prevents opioid high).	Fox et al. (2015); Tomori et al. (2014)
	**Sense of self/personal disposition in relation to addiction or addiction**	Sense of one’s self and how they perceive their individual qualities which impact MOUD preferences/attitudes, or sense of one’s addiction like how severe their addition is currently or in the past.	Interest in behavior change, self-drive, treatment readiness, willpower to not use, optimism, MOUD enhances sense of control, acknowledgement of history of addiction, perceived stability to one’s gained from MOUD, personal responsibility for one’s addiction.	Hyatt et al. (2023); Thompson and Clegg (2023)
	**Demographics**	Demographic characteristic(s) and how those impact MOUD preferences/attitudes.		
	Age	How age impacts MOUD preferences/attitudes.	Methadone perceived as unavoidable for older people, peers discouraged younger people from methadone.	Hoffman et al. (2023)
	Gender	How gender impacts MOUD preferences/attitudes.	Women shared experiences of trauma that informed sharing of their MOUD utilization, responsibilities related to motherhood informed MOUD, desire for gender-specific treatment.	Gallagher et al. (2023); Skogseth et al. (2024)
	Race	How race impacts MOUD preferences/attitudes.	Black participants were less accepting of naltrexone (vs. White).	Hyatt et al. (2023)
	**Stigma internalized**	Internalized stigma toward anything related to MOUD (MOUD broadly, specific MOUD, OUD).	Internalized stigma generally and during dosing, embarrassment.	Culbert et al. (2015); Marshall et al. (2023)
	**Substance use**	Current or previous nonprescribed substance use (e.g., opioids and beyond).		
	Continue to reduce use	Continuation of substance use while receiving MOUD treatment or continuation of substances as a reason to not receive MOUD, or MOUD as a impetus for not continuing substance use.	MOUD keeps alive during continued opioid use, MOUD types that block effects of opioids, avoidance of nonprescribed use, reducing nonprescribed use.	Havnes et al. (2014); Larney et al. (2017)
	Drug abstinence	Desire for substance abstinence as a reason to stop or start MOUD treatment, role of abstinence in MOUD type choice.	Desire to quit substances or be free from dependence, appraisal of which MOUD type would be easiest to attain abstinence.	Evans et al. (2023); Komalasari et al. (2021)
	**Withdrawal experiences and avoidance**	Withdrawal experiences in the past or fear of withdrawal lead to MOUD preferences/attitudes, including present and future avoidance.	Withdrawal induced at intake to incarceration (no MOUD or interrupted MOUD), desire to avoid withdrawal, fear of withdrawal while switching between MOUD types, anticipate worse withdrawal with more severe MOUD dependency.	Ascunce Gonzalez et al. (2024); Chappuy et al. (2021)
Interpersonal	**Peers interactions or perceptions**	Peer interactions or actions, or perceptions of MOUD or recovery that shape their perspective on MOUD and selection of MOUD.	Rejection from peer group for MOUD, tension with incarcerated peers, peers desiring selling MOUD, negative attitudes from peers toward MOUD.	Marshall et al. (2023); Stopka et al. (2024)
	**MOUD exposure via others**	Exposure to MOUD, through any means (e.g., stories, seeing in-person), via one’s peers, family, friends, or community.	Know family or friends receiving MOUD from same clinic, saw MOUD benefit others.	Nguyen et al. (2024); O'Hara et al. (2022)
	**Social and familial support**	General support networks or familial relationships (“family” or children) that bolsters or deters patient from MOUD.	General support networks support MOUD, choose to receive MOUD to win support of networks, familial pressure to find “normalcy” without MOUD. Family motivated MOUD, desire for relationships with children therefore positive or negative attitudes toward MOUD, MOUD as a tool to feel more stable for family, lack of family structure confounded post-release stability.	Frank and Walters (2021); Kaplowitz et al. (2023)
Community	**Housing**	Impact of housing access and quality on MOUD treatment linkage and engagement.	Housing instability impacted ability get treatment post-release, fear of homelessness causing MOUD discontinuation post-release, fear of MOUD impacting housing, housing facilitated MOUD engagement.	Matheson et al. (2022); (Russell et al., 2022a)
	**Interactions with legal personnel**	Interactions with the legal system, spanning all intercepts, and how those shaped attitudes toward MOUD.	Stigma from correctional staff and law enforcement, distrust toward correctional staff including toward MOUD, fear of mistreatment from correctional staff, drug court supported or coerced patients into MOUD, police harassment outside of methadone OTPs.	Evans et al. (2023); Martin et al. (2019)
	**Healthcare personnel interactions**			
	Medical staff	Personal interactions with medical system personnel and how those shaped attitudes toward MOUD.	Positive or negative interactions with medical staff, stigma or misinformation from medical providers.	Liberman et al. (2021); Nguyen et al. (2024)
	Peer recovery specialist	Personal interactions with peer recovery specialists and how those shaped attitudes toward MOUD.	MOUD supported by peer recovery specialist.	Kaplowitz et al. (2023); Skogseth et al. (2024)
	**Social service programs**	Need for social service programs to utilize MOUD.	Homeless shelter stays or hospitalizations can result in cessation of MOUD, need for social programs to maintain initiation.	Hoffman et al. (2023); Matheson et al. (2022)
	**Substance use availability and access**	Availability and access to substances in the community (not necessarily use and not the supply).	MOUD initiation due in part to lack of opioid access (not specified why opioid access was lacking), access to nonprescribed opioids during incarceration.	(Havnes et al., 2014); Treitler et al. (2022)
	**Transportation**	Role of transportation access and quality for MOUD engagement.	Poor transportation access, methadone engendered more transportation needs, not connecting to treatment due to transportation.	Martin et al. (2019); Russell et al. (2022a)
	**Treatment availability and access**			
	Access to MOUD	General access to MOUD, at all or specific types, opportunity to receive MOUD(s), or prior MOUD use generally.	COVID reduced access to clinics, MOUD should be accessible, planful of access prior to incarceration, incarceration as first time to access MOUD, previous use of MOUD (generally), previous use of MOUD impacting future use.	Dorgay et al. (2022); Treitler et al. (2022)
	Nonprescribed treatment	Nonprescribed MOUD use and/or development of one’s own treatment plan using MOUD.	Use of nonprescribed MOUD for withdrawal management, use prescribed MOUD differently for desired effects, desire to illegally purchase over receive from clinic, previous nonprescribed or misuse of MOUD.	Chappuy et al. (2021); Gryczynski et al. (2021)
	Adjunctive services	Adjunctive services during MOUD treatment perceived as essential to success or a preference.	Adjunctive resources broadly, educational classes (e.g., family classes), counseling or psychosocial support, recovery meetings, residential treatment.	Finlay et al. (2020); Skogseth et al. (2024)
	Dosing administration	Administration of MOUD including whether the administration is discreet, how frequent it is, and the route of administration.	Injectable as more discreet, administration frequency feed up time with less clinic visits, route of administration was negative (e.g., needles, bad taste) or positive (e.g., mechanism of action, no potential for misuse).	Stopka et al. (2024); Treitler et al. (2022)
	Dose protocol	Details of the dose protocol, specifically, that impact the treatment experience.	Dosing concerns (unspecified), imprecise dose due length of time sublingual held under tongue, dose reduction too fast.	Kaplowitz et al. (2022b)
	Dosing take home	Take-home doses and the impact on treatment experience.	Take-homes facilitated MOUD.	Russell et al. (2022a)
	MOUD program rules (community)	MOUD program rules in the community that shape the treatment experience.	Dislike for regular urine screens and daily dosing (methadone), like for regular urine screens and daily dosing (methadone), wait-times to engage in MOUD/delays in care, negotiation between clinic and other systems requirements, high demands on individuals and admissions requirements like paperwork, administrative obstacles.	Kaplowitz et al. (2022b); Larney et al. (2017)
	MOUD program rules (incarceration)	MOUD program during incarceration that shape the treatment experience.	Delays in MOUD from intake, restrictions to MOUD during incarceration (serving short sentences only, other medical conditions), restrictive policies made methadone seem dangerous, if diverted a MOUD required to receive injectable, daily dosing (methadone) or not (injectable), offered MOUD at initiation, eligibility miscommunication, dislike for timing of dosing, dose tapers too rapid, delivery of methadone in specific units was disliked.	Brinkley-Rubinstein et al. (2019); Maradiaga et al. (2016)
	Social and political within or around clinic setting	Social and political climate within the clinic setting that shape the treatment experience.	Fears of running into peers at clinic, violence around clinic, clinic as change in social context (community); methadone as social risk, fear of harassment from others due to clinic involvement, violence avoided due to MOUD receipt (incarceration); medical info not exchange among relevant parties.	Hayashi et al. (2017); Mukherjee et al. (2016)
	**Treatment quality**			
	Incarceration as detox	Incarceration in jail or prison as a detox period since there was no MOUD available (entered incarceration receiving MOUD or opioids).	Incarceration as opportunity for detox from any opioid or MOUD, easier to ensure the detox during incarceration.	Marshall et al. (2023); Swartz et al. (2022)
	Treatment history during incarceration	Prior MOUD treatment experiences during incarceration, including experiences of withdrawal (detox).	Extreme feelings of restriction during incarceration, poor experiences with MOUD treatment while incarcerated, lack of MOUD availability at other carceral facilities, strong aversion to methadone due to painful withdrawal during incarceration, desire for MOUD during incarceration due to past experiences without it (fear of withdrawal).	Hayashi et al. (2017); Maradiaga et al. (2016)
	Sufficient dose	Sufficient MOUD dose and the impact on treatment experience.	Too low dose leads to overdose risk, too low dose does not satisfy cravings/pain/withdrawal symptoms.	Havnes et al. (2014); Michener et al. (2024)
	Tailored program	Tailored treatment programs to the individual.	Preference for tailored program like gender-specific, dose reductions for individual needs.	Maradiaga et al. (2016); Skogseth et al. (2024)
	Interruptions	Interruptions in MOUD treatment that shaped perceptions, both perceived and experienced.	Injectable as less interruption risk, interruptions between incarceration and community provider connection, MOUD not available during incarceration leading to interruption, concerns about missing doses, interruptions can and have been withdrawal-inducing.	Martin et al. (2019); Matheson et al. (2022)
Structural	**Economic conditions**			
	Costs	Costs associated with their decision to receive MOUD and shaped decision making, such as costs associated with MOUD itself or costs associated with substance use.	MOUD saved money, MOUD costs money, MOUD led to avoidance of money spent on substances or substance-related debts.	Larney et al. (2017); Liberman et al. (2021)
	Employment	Role of employment as a facilitator or deterrent of MOUD utilization and how it shapes preferences to/to not receive MOUD.	MOUD interference with work schedules, having a job facilitated continued MOUD receipt, desire for employment as motivator for MOUD.	Vail et al. (2021); Velasquez et al. (2019)Vail et al. (2021)
	**Insurance**	Health insurance and the influence it exerts on patient’s perception of ease of care receipt.	Concerns about re-enrollment post-release deterred MOUD plans, concerns about potential costs of MOUD.	Russell et al. (2022a); Treitler et al. (2022)
	**Legal policies**			
	Legal administration distrust or fear	Expressed distrust of fear toward criminal-legal systems or upper administration including distrust toward correctional institution administrators and fear of child protective services.	Fear of child removal from child protective service or desire to want children home so use of MOUD as motivator, fear of MOUD jeopardizing parental rights, losing child custody negatively impacted MOUD engagement, interest in MOUD despite pressure from correctional administrators, distrust in MOUD due to correctional administrators.	Gallagher et al. (2023); Marshall et al. (2023)
	Legal pressure	Perceive pressure exerted from the criminal-legal system that compelled patients to or to not receive MOUD.	Ordered or suggested to MOUD to avoid legal sentencing, intimidating surveillance from correctional staff while dosing, illegality of opioids pushed patient toward MOUD.	Evans et al. (2023); Frank and Walters (2021)
	Documentation needs	Any needs or barriers related to documentation that influence perceptions of MOUD and MOUD access.	Documentation and language barriers, not having appropriate documentation to initiate treatment.	Ascunce Gonzalez et al. (2024); Russell et al. (2022a)
	Parole probation conditions	Conditions of parole or probation encourage or discourage MOUD receipt and attitudes toward MOUD.	Mandated MOUD treatment, fear of repercussions for MOUD utilization, complex communication processes between agencies post-release.	Vail et al. (2021); Velasquez et al. (2019)
	**Criminal-legal system interactions**			
	Dependent on criminal-legal intercept	How perceptions or decisions were formed based upon a valuation of what was needed for an individual at a given criminal-legal intercept (e.g., “while incarcerated” or “upon release from incarceration”).	Address basic needs prior to MOUD post-release, lower endorsement of methadone during incarceration (vs. post-release), different intentions with methadone during incarceration (vs. use of MOUD in community), elect to receive a MOUD during the post-release period due to known overdose risk	Brinkley-Rubinstein et al. (2019); Velasquez et al. (2019)
	Legal involvement (unspecified)	Unspecified involvement with the criminal-legal system and how this impact MOUD preferences/attitudes.	Having criminal-legal involvement history associated with preference for residential vs. MOUD treatment.	Stein et al. (2015)
	Incarceration history	The influence of past incarcerations (not matter the duration or experience) on MOUD preferences/attitudes.	Higher willingness for MOUD vs. those without history, desire to never return to jail, prior poor experiences while incarcerated.	Frank and Walters (2021); Zelenev et al. (2018)
	Life responsibilities	Life responsibilities that motivated interest in MOUD.	Non-daily dosing frees up time, daily dosing interferes with other responsibilities, setting personal goals for stability with MOUD.	Ascunce Gonzalez et al. (2024); Gallagher et al. (2023)
	Planning post-release	Desire to or not to receive MOUD post-release, specifically.	Interest in MOUD post-release for health benefits and time to adjust post-release, need to meet other basic needs them establish MOUD post-release, negative impact of rapid dose reductions during incarceration on the post-release period.	Larney et al. (2017); Maradiaga et al. (2016)
	Planning post-release (facilitated)	Post-release planning including MOUD that was facilitated by a system like a re-entry specialist at a carceral facility.	Re-entry services facilitated MOUD, desire to stay at OTP upon release, residential supports post-release were variable and impacted release date, essential to establish logistics like MOUD, ID, and housing prior to release to keep people stable.	Hoffman et al. (2023); Treitler et al. (2022)
	**Stigmas and related ideologies**			
	Drug free ideology	Influence of a substance free ideology (i.e., value in not using substances, such as not using MOUD for fear of MOUD being substances) on MOUD preferences/attitudes, focus on philosophy rather than just not using substances.	MOUD as continued substance use or poison, perception of growing out of substance use.	Kaplowitz et al. (2022b); Velasquez et al. (2019)
	MOUD criminalization	Influence of MOUD criminalization (i.e., experienced or feared legal interactions due to MOUD utilization or receipt) on MOUD preferences/attitudes.	Fears of arrest deterring methadone receipt.	Tomori et al. (2014)
	Stigma: criminal record	Influence of stigma toward criminal record on MOUD preferences/attitudes.	Interferes with ability to continue treatment, stigma toward methadone compounded.	Havnes et al. (2014); Nguyen et al. (2024)
	Stigma: methadone	Influence of stigma toward methadone (specifically) on MOUD preferences/attitudes.	Methadone considered a drug, methadone as associated with socially undesirable health (e.g., HIV, lazy) and social (e.g., impoverished, abusing system) conditions, desire to be “normal” and not receiving methadone, general stigma around methadone clinics.	Larney et al. (2017); Velasquez et al. (2019)
	Stigma: MOUD	Influence of stigma toward MOUD (not specifically methadone) on MOUD preferences/attitudes.	Demeaning dosing experience, general stigma from others.	Liberman et al. (2022); Polonsky et al. (2016)
	**Substance use supply**	Perceptions of what is in the substance supply and how this impacts MOUD preferences/attitudes.	Fears of fentanyl in the substance supply.	Kaplowitz et al. (2022a); Vail et al. (2021)

MOUD =  medications to treat opioid use disorder; ID =  identification; OTP =  opioid treatment program; HIV =  human immunodeficiency virus; HCV =  hepatitis C virus; OUD =  opioid use disorder

⁎=if more than two articles were coded with a given code, the two example articles were randomly selected.

**Table:** 

Theme	Category	Code	MOUD	MTD	BUP	BUP-XR	NTX-XR

Individual	MOUD cognitive						
		Beliefs	1	2	2	1	
		Knowledge					1
		Intentions	3	2			
	MOUD physiologic response						
		Euphoria			1		
		Side effects		1			
	Sense of self/personal disposition			3			
	Demographics						
		Age		1			
	Substance use						
		Continue or reduce use	1	2	1		
	Withdrawal experiences and avoidance			1	2		
Interpersonal	Social and familial support			1			
Community	Legal personnel interactions			1			
	Substance use availability and access		1				
	Treatment availability and access						
		Access to MOUD	2				
		Nonprescribed MOUD			2		
		Dosing administration				1	
		MOUD program rules	2		1		
		Social/political in clinic	1				
	Treatment quality						
		Incarceration as detox		1			
		Treatment history during incarceration	1	1	1		
		Tailored program	1				
Structural	Legal policies						
		Legal pressure		1			
		Parole probation conditions	2				
	Criminal-legal systems involvement						
		Dependent on criminal-legal intercept	3				
		Incarceration history	2				
		Planning post-release	1				
						
	Stigmas and related ideologies						
		Drug criminalization		1			
		Stigma: substance use		2			
	Substance use supply		1	1			

MOUD =  medications to treat opioid use disorder; NTX-XR =  naltrexone extended release; BUP =  buprenorphine orally administered; BUP-XR =  buprenorphine extended release

Note: Counts represent the number of papers where the factor is present.

## Data Availability

Not applicable. All data for this scoping review are published manuscripts.

## Appendix B Full Search Protocol

Preliminary PubMed search conducted on September 12, 2024.
